# Case report: Zanubrutinib-induced dermatological toxicities: A single-center experience and review

**DOI:** 10.3389/fonc.2022.941633

**Published:** 2022-07-25

**Authors:** Lingling Wang, Jiao Tang, Jun Feng, Yongfen Huang, Yuexin Cheng, Hao Xu, Yuqing Miao

**Affiliations:** ^1^ Department of Hematology, The First people’s Hospital of Yancheng, The Yancheng Clinical College of Xuzhou Medical University, Yancheng, China; ^2^ Department of Neurology, The First people’s Hospital of Yancheng, The Yancheng Clinical College of Xuzhou Medical University, Yancheng, China

**Keywords:** zanubrutinib, dermatological toxicity, maculopapule rash, papulopustular rash, skin infection, epithelial growth factor receptor

## Abstract

Zanubrutinib, a next-generation non-covalent Bruton’s tyrosine kinase (BTK) inhibitor, shows great efficacy in the treatment of B cell malignancies. Some patients may experience a series of side effects after the treatment of zanubrutinib. Grade 4 dermatological toxicities are rare, which present as severe rash and skin infection. Herein, we retrospectively reported the grade 4 dermatological toxicities of zanubrutinib in three consecutive patients. They were treated with zanubrutinib 160 mg twice daily orally. One patient was diagnosed with Primary Breast Diffuse Large B-cell Lymphoma(PB-DLBCL) and two patients were diagnosed with Chronic Lymphocytic Leukemia(CLL). Within one month after zanubrutinib treatment, all three patients developed grade 4 dermatological toxicities, including bruising, maculopapular rash, petechiae, ecchymosis, hemorrhagic blister, acne-Like rash, papulopustular rash, and skin infections. Zanubrutinib was discontinued in two patients due to unacceptable dermatological toxicities. Safety data from pre-licensing clinical trials showed that zanubrutinib-related side effects were frequent but well tolerated. To date, no severe dermatological toxicities were reported. The majority of patients can be relieved with symptomatic treatment, but a very small percentage of patients may face discontinuation of the drug.

## Introduction

BTK is a non-receptor tyrosine kinase in the Tec(transient erythroblastopenia of childhood) family that is mainly expressed in hematopoietic stem cells such as B cells, monocytes, macrophages, and basophils. Since BTK plays an important physiological function in the B-cell receptor and FcγR-mediated signaling pathway, BTK has become an important therapeutic target for the treatment of B cell lymphoma. There are currently five BTK inhibitors approved for marketing. Zanubrutinib is a highly selective BTK inhibitor that forms covalent bonds with cysteine residues at the BTK active site, thus inhibiting BTK activity. Zanubrutinib, a representative drug of the second-generation BTK inhibitors, still inhibits EGFR, HER2, ITK, JAK3, TEC, BMX, and BLK, although its off-target effect is significantly reduced compared to first-generation BTK inhibitors (1). Dermatological toxicities are off-target effects and are supposed to relate to EGFR inhibition, which frequently appears during the first year of treatment. The overwhelming majority can be improved over time, so reports about dermatological toxicities related to zanubrutinib are diminished as the duration of treatment increases (2). Herein, we reported our experience with zanubrutinib-induced dermatological toxicities in three consecutive patients with B cell malignancies. Once grade 4 dermatological toxicities occur, topical hormonal medication and anti-allergic treatment are recommended. Some patients require empirical anti-inflammatory treatment. In severe cases, consultation with a dermatology specialist is recommended.

## Methods

Three consecutive patients were diagnosed with B cell malignancies in the Department of Hematology of The First people’s Hospital of Yancheng. Bone marrow aspiration and image logical examination were done in all patients to confirm the diagnosis. One patient diagnosed with PB-DLBCL, received a regimen containing rituximab, lenalidomide, and zanubrutinib. The other two patients were diagnosed with relapsed CLL and treated with oral zanubrutinib. All patients were treated with zanubrutinib 160 mg twice daily continuously, until disease progression or unaccepted toxicities.

## Results

### Case 1

**Figure 1:**
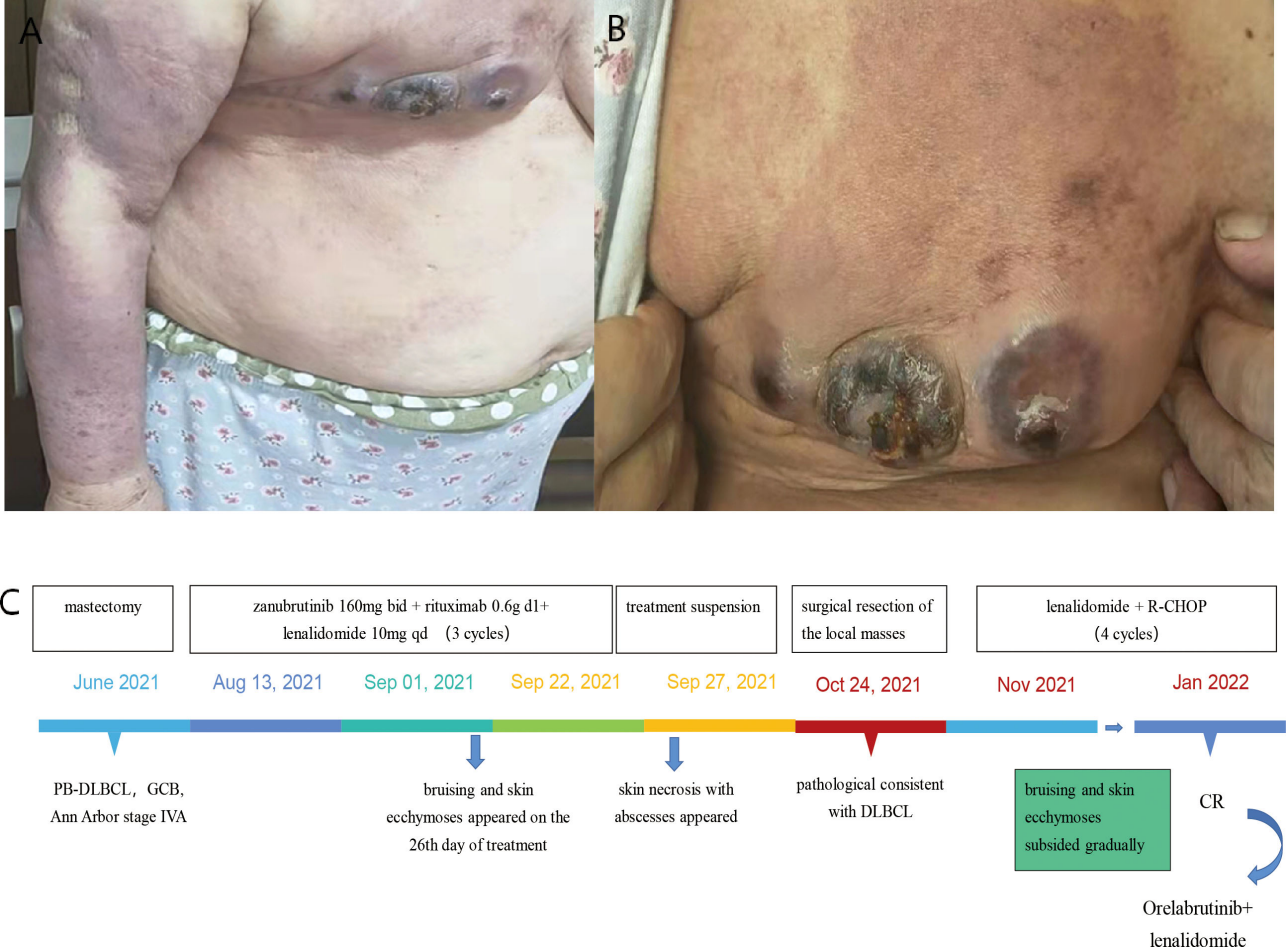
Case 1 : A 71-year-old woman with PB-DLBCL. **(A)** swelling of the right upper extremity. **(B)** bruising and skin ecchymoses on the right upper extremity and the right anterior chest wall. **(C)** The time line of this patient.

A 71-year-old female patient was admitted to our hospital because of her inadvertent discovery of a right breast mass in June 2021. The patient denied any relevant personal or family history. Palpation examination revealed an irregular mass with a medium texture. Mastectomy was performed in the Department of Surgery of our hospital. The pathological report revealed the lesion diffuse large B-cell lymphoma(DLBCL). The pathological immunohistochemical staining were as follows: CD10+, CD138-, CD20+, CD21-, CD23-, CD3+, CD38+, CD45RO+, CD5-, CD79α+, MUM1+, c-Myc+, CyclinD1-, Bcl2+, Bcl6+, Ki67(+:80%), SOX-11-, PAX5+, Kappa-, Lambda+, CK-P-, Vim+. The whole-body positron emission tomography-computed tomography (PET-CT) scan and bone marrow aspiration were performed to evaluate the condition. She was finally diagnosed as PB-DLBCL, germinal center B-cell type (GCB), Ann Arbor stage IVA. The Patients received three cycles of targeted therapy including zanubrutinib, rituximab, and lenalidomide in August and September of 2021. Her blood profile was monitored three times a week. After 26 days of treatment, she developed bruising and skin ecchymoses on the right upper extremity and the right chest wall, but she continued to take the drug. After three cycles of chemotherapy, there was no significant improvement in bruising and skin ecchymoses. More severely, swelling of the right upper extremity was much more obvious. Zanubrutinib was withheld as a suspected cause of dermatological toxicity, and third-generation cephalosporin therapy was undertaken. Furthermore, the patient developed three severe skin necrosis with abscesses in the right chest wall, which evolved into cheese-like changes over time. The final decision was made to perform surgical resection of the local masses after multidisciplinary discussion, and the pathological findings were consistent with lymphoma. The pathological immunohistochemical staining were as follows: CD3-, CD5-, CD20+, CD79a+, CD21-, CD23-, CD10+, CD15-, CD30-, CyclinD1-, MUM1+, Bcl-2+, Bcl-6+, Ki67+, PAX5+. This suggested that necrosis of the right chest wall skin was associated with disease progression. Reexamination of CT indicated the patient’s progress, then we switched the regimen to lenalidomide, along with rituximab-cyclophosphamide, hydroxydaunomycin, oncovin, and prednisone(R-CHOP) chemotherapy. The bruising and skin ecchymoses on the right upper extremity subsided gradually in the following months. After four cycles of R-CHOP chemotherapy, PET-CT showed that the patient achieved complete remission. The patient was given orelabrutinib plus lenalidomide as a maintenance regimen. No similar dermatological toxicities occurred again during any of these periods (**Figure 1**).

### Case 2

**Figure 2:**
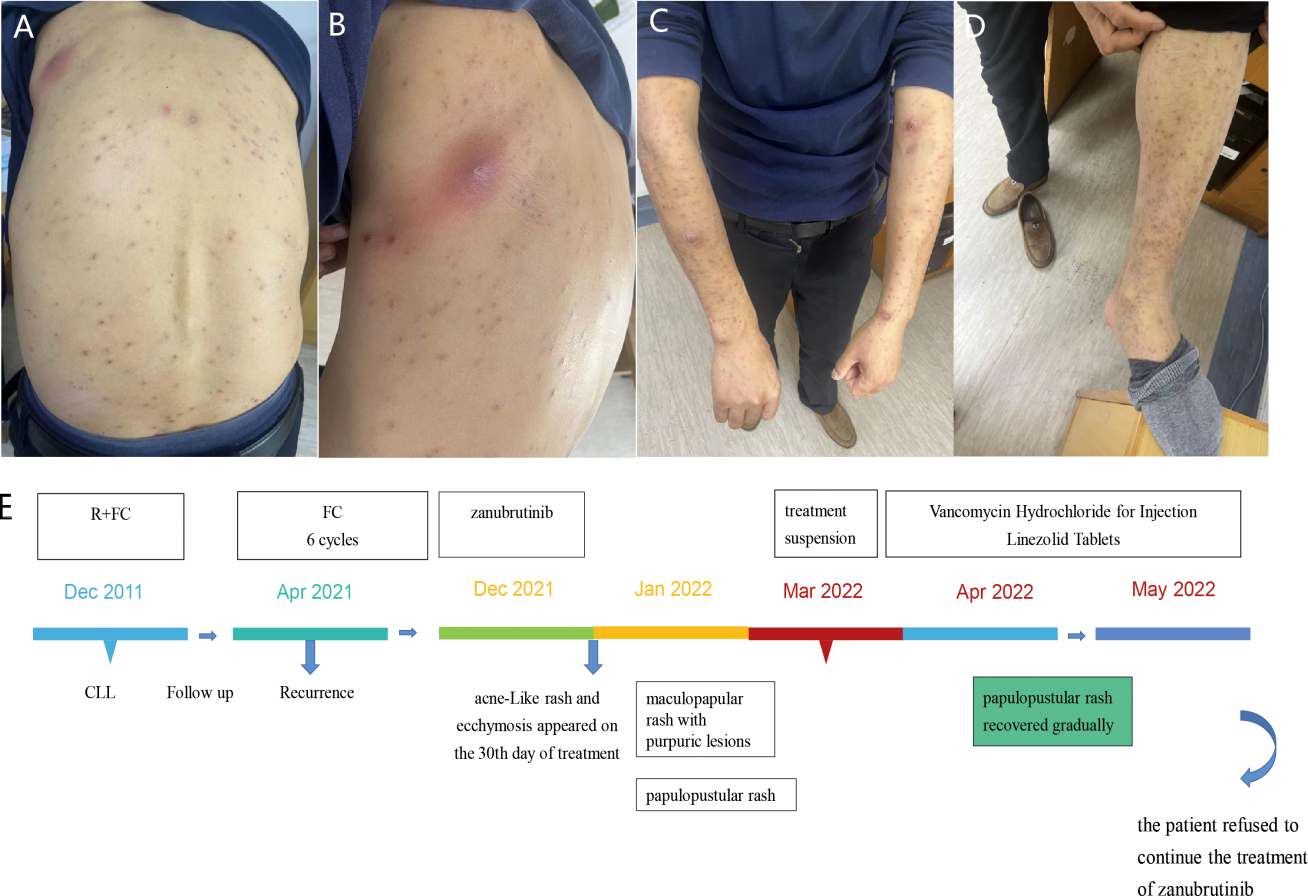
Case 2 : A 56-year-old man with CLL. **(A, C, D)** difused and severe maculopapular rash with purpuric lesions on the trunk, upper extremity and lower extremity. **(B)** necrotizing folliculitis with suspected Staphylococcus aureus superinfection on the back. **(E)** The time line of this patient.

A 56-year-old man was diagnosed with CLL at the age of 45 and was previously treated with rituximab, fludarabine, and cyclophosphomide chemotherapy. In April 2021, the patient presented with progressive splenomegaly, lymphadenopathy, peripheral blood thrombocytopenia, and lymphocytosis. Fludarabine and cyclophosphamide treatment was restarted. The patient achieved partial response quickly and received zanubrutinib as maintenance treatment. After 30 days of treatment of zanubrutinib, acne-Like rash and ecchymosis ran over the whole body, and diffused maculopapular rash with purpuric lesions gradually appeared. The patient developed a papulopustular rash involving the nape of the neck, trunk, axilla, limbs, and groin area after two months of treatment. He did not show any symptoms of fever or systemic allergy. Bacterial cultures were taken several times, but the results were all negative. We had suggested skin biopsy several times, but the patient refused skin biopsy. Due to our experience, the special skin rash was related to the side effect of zanubrutinib. After careful consideration, zanubrutinib was stopped. Treatment with topical corticosteroids was proposed and the effect was not obvious. We empirically treated the patient with vancomycin according to the local epidemiological characteristics, and the patient’s skin erythema and rupture did improve significantly after vancomycin treatment. On our advice, the patient continued oral linezolid tablets for maintenance treatment. The patient is currently in the follow-up phase and the generalized rash has significantly subsided and improved, but the patient refused to continue the treatment of zanubrutinib (**Figure 2**).

### Case 3

**Figure 3:**
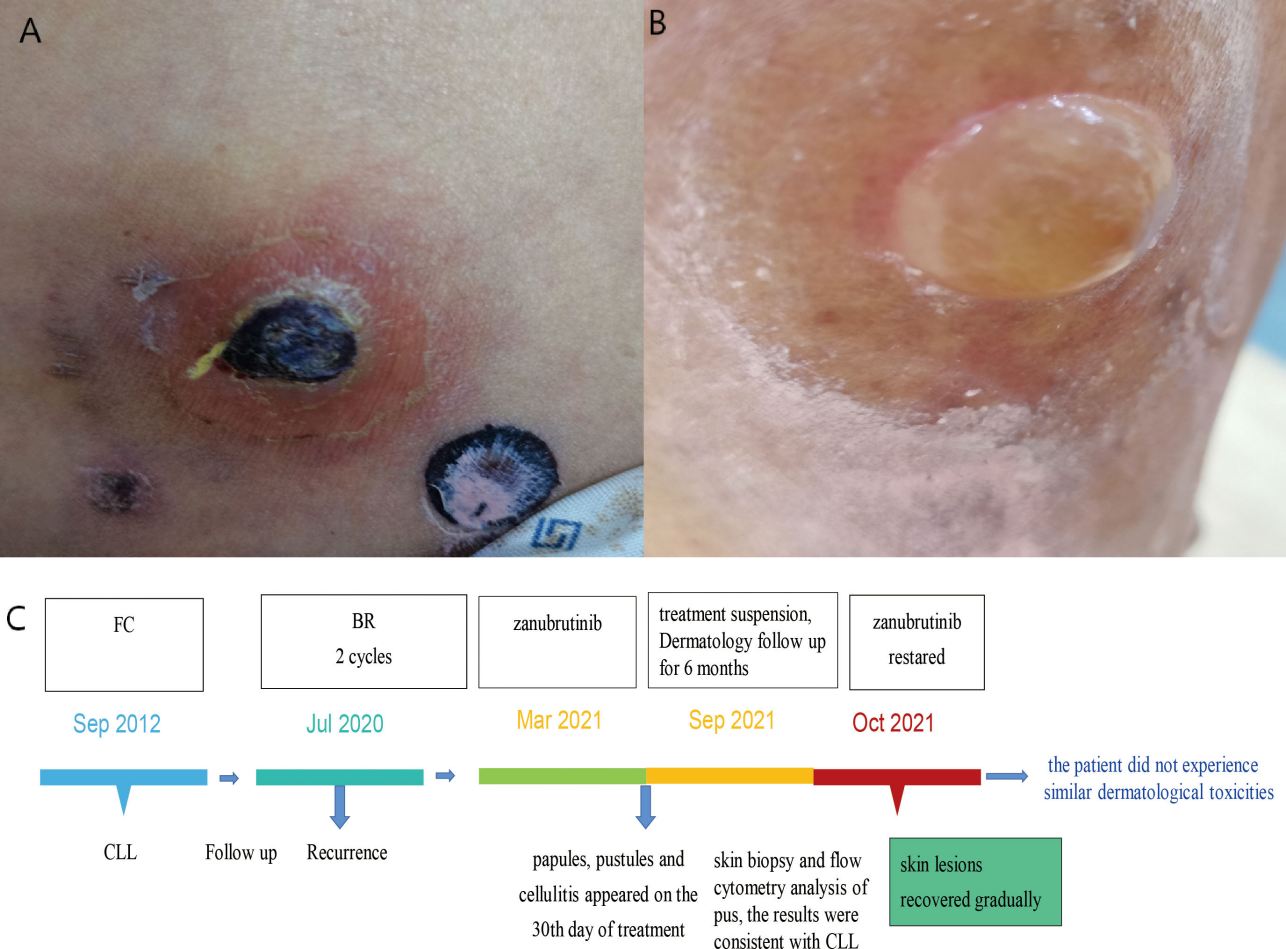
Case 3 : A 69-year-old man with CLL. **(A)** cutaneous ecchymosis with central necrosis. **(B)** pustules on the left lower extremity. **(C)** The time line of this patient.

In July 2020, a 69-year-old male patient was admitted with a relapse of CLL, diagnosed 8 years ago, initially treated with FC(i.e., fludarabine and cyclophosphamide) regimen chemotherapy and discontinued in March 2013. He had severe anemia, lymphadenopathy, hyperleukocytosis with lymphocytosis, and mild thrombocytopenia. Peripheral blood flow cytometry analysis was consistent with CLL, bone marrow studies showed massive and diffuse infiltration with clonal small B lymphocytes. He was treated with BR(i.e., rituximab and Bendamustine) regimen chemotherapy and received a therapeutic response. The patient achieved partial remission after two cycles of treatment, then switched to zanubrutinib as maintenance therapy. One month after treatment, multiple lesions began to appear on his left lower extremity, initially presenting as papules, which later developed into pustules and cellulitis. The patient was followed by Dermatology Department for clearing the wound. Topical fusidic acid cream was used as an anti-inflammatory agent, and compound calamine lotion was used to reduce itching. However, the skin lesions were persistent over his left lower limb for about six months. The patient had necrotic tissue removed from the left lower extremity wound in the Department of Dermatology, and the skin biopsy results suggested that the tissue was infiltrated by acute and chronic inflammatory cells with necrosis. We also retained pus from this area for flow cytometry analysis and the results were as follows: CD19+, CD5+, CD20+, CD25+, CD23, cBcl-2+, cKappa-, sKappa-, sLambda-, CD138-, CD10-, CD38-, sIgM-, CD102-, CD11c-, FMC-7-, CD22-. Treatment and follow-up in the dermatology department were carried out for more than 6 months and the skin eventually healed well. On our advice, the patient was reintroduced to oral zanubrutinib in October 2021, after which the patient did not experience similar dermatological toxicities (**Figure 3**).

## Discussion

Dermatological toxicities are among the most common toxicities of BTK inhibitors (3). Bruising, skin ecchymoses, eczema-like rash, acne-like rash (folliculitis), pityriasis rosea-like rash, and panniculitis are the typical dermatological adverse events. Nail changes include brittleness onycholysis, onychorrhexis, onychoschizia, koilonychia, or trachyonychia paronychia, and subungual splinter hemorrhages. Hair changes are also common, including alopecia, and the hair follicle changes from curly to straight. Stomatitis is not rare. Other dermatological toxicities include neutrophilic dermatosis, skin carcinomas, autoimmune skin disorders, xerosis, peripheral edema, and eosinophilic dermatosis of hematologic malignancy (EDHM).

Here, we retrospectively reported three cases of dermatological toxicities induced by zanubrutinib. All three patients developed grade 4 skin adverse effects, which lead to a suspension of treatment in two patients. The first patient was diagnosed with PB-DLBCL, received combination chemotherapy containing zanubrutinib, lenalidomide, and rituximab, and developed widespread bruising and skin ecchymoses on the right chest wall and right upper extremity on day 26 of treatment. All three medicines have the potential to produce dermatological toxicity, and we took into account this patient’s zanubrutinib-related dermatological toxicity for the following reasons. When given rituximab for the first time, some people may develop a rash allergy. This is usually a transitory reaction that can be alleviated by using anti-allergic medications and slowing down the titration rate. On the day of rituximab treatment, the patient did not experience any usual adverse symptoms such as rash, pruritus, chest tightness, or dyspnea. The dermatological toxicities did not occur when the patient had chemotherapy with the R-CHOP regimen and lenalidomide, and the timing of the skin reaction in this patient does not match the time point of rituximab treatment. Therefore, we concluded that the rash in this patient was not related to rituximab or lenalidomide. In contrast, skin necrosis at the right breast incision was confirmed by biopsy to be associated with lymphoma progression. PET-CT corroborated the recurrence of the disease. Both the second and third patients were diagnosed with relapsed CLL, received FC regimen chemotherapy and achieved remission, and were maintained on zanubrutinib without taking other drugs that could cause dermatological toxicity during maintenance therapy. Therefore, the dermatological toxicities in these two patients were related to zanubrutinib.

To date, there are no reported cases of dermatological toxicities secondary to zanubrutinib in China. We report the first three cases of dermatological toxicities due to zanubrutinib. Our team hopes these cases could raise clinical awareness regarding zanubrutinib-induced dermatological toxicities and the importance of drug withdrawal in the event of dermatological toxicities. We review the underlying mechanisms of dermatological toxicities, and the incidence of zanubrutinib-associated dermatological toxicities, and propose our experience in managing them.

### Underlying mechanisms of dermatological toxicities

It has been postulated that the direct binding to both BTK and other ‘off-target’ kinases leads to BTK inhibitors-related dermatological adverse events. Zanubrutinib has overwhelming advantages in optimizing BTK inhibition and minimizing off-target inhibition of alternative kinases (Tec, ITK, EGFR, SRC-family kinases) (4). EGFR and downstream signaling pathways are involved in numerous key biological processes such as cell proliferation, differentiation, migration, and apoptosis. In skin tissues, EGFR receptors are expressed on keratinocytes, which are distributed in the basal and suprabasal layers of the epidermis as well as in the outer layer of the hair follicle. The EGFR pathway regulates the normal growth and differentiation process of the epidermis, stimulating epidermal growth, inhibiting differentiation, and accelerating wound healing. Blocking the EGFR pathway in the skin can lead to a series of inflammatory reactions, and thus manifest the corresponding skin adverse effects (5). EGFR-TKI could evoke dermatological and gastrointestinal toxicities through block down epidermal growth factor signals. Dermatological toxicities involved rash acneiform, skin fissure, and xerosis, which are related to pruritus (6). Acne-like rash with erythematous papules or pustules centered on hair follicles is the most common clinical manifestation of adverse skin reactions. With few subjective symptoms, no effect on daily life, and no secondary infection, grade 1 adverse reactions are limited to the head, face, and upper trunk. Grade 2 side effects along with minor subjective symptoms, little impact on daily life, and no signs of secondary infection. To adverse reactions of grade 3/4, the subjective symptoms are severe, causing significant disruption in daily life and the risk of secondary infection. Premature differentiation, inflammation, apoptosis, skin atrophy, telangiectasia, and photosensitivity are all side effects of EGFR inhibition (7). Although zanubrutinib is a highly selective BTK inhibitor, it also inhibits cell cycle progression and increases apoptosis by acting on other kinases such as EGFR. The most likely mechanism of zanubrutinib-induced skin rash appears to be off-target inhibition of EGFR. Inhibition of c-kit and platelet-derived growth factor receptors is another mechanism proposed for zanubrutinib-induced drug eruption. Iberri hypothesized that some of these rash types, particularly those that appeared within the first month of treatment, could be related to the transient hyperlymphocytosis associated with BTK, which is caused by CLL cells egressing from lymph nodes and spleen (8).

We believe that the dermatological toxicities seen in these three patients were not the same phenomenon as CLL-associated insect bite-like reactions. Insect bite-like reaction, also known as eosinophilic dermatitis associated with hematologic malignancies, is a nonspecific skin reaction to a hematologic disease, which results in an altered immune response and increased secretion of TH2 cytokines (IL4 and IL5) that stimulate the development of eosinophilic skin infiltrates (9, 10). All three patients denied a history of any insect stings, food allergies, and drug allergies. The first patient was newly diagnosed with DLBCL and did not undergo any treatment that could have induced dermatological toxicities before zanubrutinib-based targeted therapy. The second and third cases were diagnosed with relapsed CLL and did not experience any degree of dermatological toxicities during previous chemotherapy and follow-up. All three patients developed varying forms of dermatological toxicities within one month of zanubrutinib treatment, and we do not believe that these were coincidences. Dermatological toxicities associated with ibrutinib have been reported, and most of these dermatological toxicities occur within 1 year after ibrutinib, and the incidence decreases gradually with time. The three patients observed in our center had common features with previous cases reported in the literature. Compared to ibrutinib, zanubrutinib is more precisely targeted and has fewer toxic side effects. As a result, the incidence of dermatological toxicities associated with zanubrutinib is much lower, and there are very few reports in the literature. There are three kinds of BTK inhibitors currently in use in our center, namely ibrutinib, zanubrutinib, and orelabrutinib. Based on the results of the follow-up, only these 3 patients have experienced grade 4 dermatological toxicities so far. This is the purpose of our study as a way to draw the attention of investigators and to present our treatment experience to better manage the adverse reactions of BTK inhibitors.

#### Incidence of zanubrutinib-associated dermatological toxicities

The efficacy and safety of zanubrutinib were evaluated in the following clinical trials (**Table 1**). Safety data were obtained from five multicenter studies that enrolled a total of 394 patients who received zanubrutinib. No grade ≥3 dermatological toxicities were reported. No deaths (all R/R patients) were attributed to dermatological toxicities. Almost all patients (98%) reported ≥1 Treatment-Emergent Adverse Events(TEAE). Dermatological toxicities reported in ≥10% of the study population were rash, bruising, petechiae, purpura, contusion, and rash maculo-papular (11).

**Table 1:** Incidence Of Zanubrutinib-Associated Dermatological Toxicities.

Clinical Trial	Phase	B-Cell Malignancies	Patients, No.	Rash	Purpura	Rash Maculo-Papular	Ecchymosis
Nct03189524	1	Cll/Sll, Mcl, Wm/Lpl, Fl, Mzl, Hcl, Or Non-Gcb Dlbcl	44	10/44 (22.72%)	5/44 (11.36%)	5/44 (11.36%)	–
Nct03206918	2	Cll/Sll	91	17/91 (18.68%)	31/91 (34.07%)	1/91 (1.10%)	–
Nct03206970	2	Mcl	86	31/86 (36.05%)	–	–	–
Nct03332173	2	Wm	44	8/44 (18.18%)	8/44 (18.18%)	–	4/44 (9.09%)
Nct03053440	3	Wm	129	No Study Results Posted			

#### Management of dermatological toxicities

Multiple treatment regimens could alleviate zanbrutinib-related dermatological toxicities. Moisturizers are typically used to manage dermatological toxicities in patients with grade 1/2 skin reactions. Patients should avoid alcohol and perfume-containing products because they can dry out the skin and limit its ability to heal under stress. In patients with moderate to severe dermatological toxicities, topical steroid ointments such as hydrocortisone can be used alone or in combination with topical emollients. In cases where skin toxicity leads to infection, antibiotic ointments or systemic antibiotics must be used. In addition, multidisciplinary cooperation is recommended, and the patient is advised to visit the dermatology department for further consultation. Due to the long-term use of immunosuppressive drugs in patients with hematologic diseases, immune function is deficient and they are prone to a variety of opportunistic infections in combination. Our recommendation is to temporarily discontinue the drug if grade 4 dermatological toxicities occur to give the patient time to deal with the severe events while still allowing the patient to continue using the drug, thereby avoiding disease progression due to discontinuation.

In conclusion, physicians should be aware of the potential dermatological toxicities of zanubrutinib, which requires vigilance. With appropriate treatment, they can be managed, minimizing patient discomfort and reducing the need for therapy interruption or discontinuation.

## Data availability statement

The original contributions presented in the study are included in the article/supplementary material. Further inquiries can be directed to the corresponding author.

## Ethics statement

The studies involving human participants were reviewed and approved by Ethics Committee of The First people’s Hospital of Yancheng. The patients/participants provided their written informed consent to participate in this study. Written informed consent was obtained from the individual(s) for the publication of any potentially identifiable images or data included in this article.

## Author contributions

LW and JT conceived and designed the study. JF, YH, YC, HX collected the clinical data. LW and YM wrote the paper. All authors contributed to the article and approved the submitted version.

## Conflict of interest

The authors declare that the research was conducted in the absence of any commercial or financial relationships that could be construed as a potential conflict of interest.

## Publisher’s note

All claims expressed in this article are solely those of the authors and do not necessarily represent those of their affiliated organizations, or those of the publisher, the editors and the reviewers. Any product that may be evaluated in this article, or claim that may be made by its manufacturer, is not guaranteed or endorsed by the publisher.
